# Transfer of extensor carpi radialis brevis branch of radial nerve to anterior interosseous nerve for lower trunk brachial plexopathy after motor vehicle accident

**DOI:** 10.3171/2022.10.FOCVID2287

**Published:** 2023-01-01

**Authors:** Alison M. Westrup, Kiana Y. Prather, Xiaochun Zhao, Mark E. Stephens, Andrew H. Jea

**Affiliations:** Department of Neurosurgery, University of Oklahoma Health Sciences Center, Oklahoma City, Oklahoma

**Keywords:** anterior interosseous nerve, brachial plexus, extensor carpi radialis brevis, nerve transfer, neuropathy, radial nerve

## Abstract

The patient is a 15-year-old male who sustained injury to his right lower brachial plexus (C8–T1) in a motor vehicle accident. Six months after the injury, the patient still had persistent hand weakness and wished to regain function in his first and second digits. Transfer of the extensor carpi radialis brevis (ECRB) branch of the radial nerve to the anterior interosseous nerve (AIN) was performed to restore motor function. The patient did well after the surgery, although it may take 12–24 months for benefits to fully manifest. Pertinent surgical anatomy and techniques are highlighted in this video demonstration.

The video can be found here: https://stream.cadmore.media/rr10.3171/2022.10.FOCVID2287

## Transcript

This video will demonstrate the transfer of the extensor carpi radialis brevis branch of the radial nerve to the anterior interosseous nerve for lower trunk brachial plexopathy after a motor vehicle accident.

### 0:34 History and Presentation.

The patient is a 15-year-old boy who sustained a right lower brachial plexus injury in a motor vehicle accident. Six months after the injury, the patient still had significant weakness in his right hand with functional impairment.

### 0:48 Exam: Klumpke Palsy—Lower Brachial Plexopathy.

On examination, he had a constellation of motor and sensory deficits in his right upper extremity consistent with Klumpke palsy as a result of lower brachial plexus injury. Although his wrist flexion was affected, his wrist extension was fully intact.

### 1:04 Exam: OK Sign—Anterior Interosseous Neuropathy.

He was also unable to make the OK sign, indicating anterior interosseous neuropathy. The anterior interosseous nerve is a motor branch of the median nerve that innervates flexor pollicis longus, flexor digitorum profundus to the second and third digits, and pronator quadratus. In anterior interosseous neuropathy, the patient is unable to flex the interphalangeal joint of the thumb and the distal interphalangeal joint of the index finger when asked to do the OK sign.

### 1:37 Rationale for Procedure.

Given the sustained neuropathy and the patient’s strong desire to be able to use his thumb and index finger, we decided to transfer a motor branch of the radial nerve, namely the extensor carpi radialis brevis branch, to the anterior interosseous nerve to restore first and second digit motor function. The ECRB branch is a deep motor branch of the radial nerve and has C7 as its primary contributing nerve root.^1^ As a result, it can be affected in an extended upper brachial plexopathy but is preserved in lower brachial plexopathy, making it an appropriate donor nerve in this case.^2,3^ In addition, because ECRB serves as an accessory extensor, the ECRB nerve can be harvested without significant impact to wrist extension. The ECRB branch most commonly arises from the posterior interosseous nerve but can also arise from the radial sensory nerve or the main trunk of radial nerve directly.^4^ AIN is the largest branch of the median nerve, leaving the main trunk of the median nerve in the proximal forearm just distal to the pronator teres. The nerve roots to the AIN are C8 and T1; therefore, the AIN is implicated in a lower trunk brachial plexopathy.

### 2:48 Role of EMG and Imaging.

Preoperative electromyography (EMG) and imaging can be used as extensions to the physical exam and aid in surgical decision-making. EMG can be used to assess muscle denervation, which may provide additional diagnostic value. Preoperative or intraoperative EMG can also be used to assess donor nerve function and may predict nerve transfer outcomes.^5^ MRI and ultrasound imaging can provide morphological details of the nerve injury. Even though these methods of evaluation may be useful, intraoperative stimulation is paramount in guiding the surgery.

### 3:27 Patient Positioning.

The patient is positioned supine on the surgical table with his right arm abducted on a hand table.

### 3:36 Incision.

This operative video shows an orientation at the top left of the screen. The patient is in the supine position. We created a lazy-S incision on the ventral surface of the forearm to gain access to both the ulnar and radial aspects.

### 3:50 Dissection and Identification of Major Vessels.

We use Bovie electrocautery to quickly get through the skin and soft tissue to the level of the fascia of the muscles of the forearm and the cephalic vein.

The cephalic vein is skeletonized and controlled with a vessel loop. The cephalic vein is then retracted radially.

The radial artery is identified next, and a vessel loop is placed. The radial artery is then likewise retracted radially.

### 4:31 Identifying the AIN.

The free edge of the pronator teres is identified and rolled toward the ulnar side with a handheld retractor. The median nerve is found under the free edge of the pronator teres.

The median nerve is followed proximally and then tagged with a vessel loop. The anterior interosseous branch is identified at the upper third of the forearm and followed proximally. Of note, the anterior interosseous nerve is the only branch of the median nerve that courses toward the radial side.

Here, the nerve stimulator, set at 20 mA, does not elicit a response in the AIN, showing the severity of the injury. The movement of the nearby muscles is from electrical spread.

### 5:38 Identifying the ECRB Branch.

Next, we turn our attention to the radial nerve. The most easily identifiable branch of the radial nerve is the radial sensory nerve. It is a pure sensory nerve and does not stimulate. Here, the fat pad underneath the radial sensory nerve vigorously stimulates at 0.5 mA, indicating a motor nerve is in close proximity.

We dissect deeper through the fat pad to identify and tag the extensor carpi radialis brevis branch of the radial nerve. The ECRB can be differentiated from the other major branch of the radial nerve, the posterior interosseous nerve (PIN), as ECRB runs parallel to the radial sensory nerve, while PIN runs deep and obliquely.

### 6:29 Dissection of the Nerves.

We follow the principle of nerve transfer surgery. Dissect as proximal as possible for the recipient nerve and as distal as possible for the donor nerve.

We place a vascular clip as distal as possible to prevent bleeding from arterioles and venules surrounding the nerve. Notice that a live nerve causes the muscle to jump as it is clipped. The donor ECRB is then divided.

### 6:53 Coaptation of the Nerves.

The cut ends of the AIN and ECRB are brought in close apposition over a rubber background.

Tension-free coaptation is accomplished through the application of a drop or two of fibrin glue. The fibrin glue is then left to harden, and the rubber background is slipped out. We prefer fibrin adhesive coaptation over suturing because it decreases operative time, mitigates neuroma formation, and provides similar functional outcomes as suturing.^6^

### 7:35 Outcome.

There was no intraoperative or postoperative complication. The patient did well after the surgery, although it may take 12–24 months for the benefit of the procedure to fully manifest.^7,8^

## Disclosures

The authors report no conflict of interest concerning the materials or methods used in this study or the findings specified in this publication.

## Author Contributions

Primary surgeon: Jea. Assistant surgeon: Stephens. Editing and drafting the video and abstract: Jea, Westrup, Prather, Zhao. Critically revising the work: Jea, Prather, Stephens. Reviewed submitted version of the work: Jea, Westrup, Prather, Stephens. Approved the final version of the work on behalf of all authors: Jea. Supervision: Jea.
